# A novel insight into neurological disorders through HDAC6 protein–protein interactions

**DOI:** 10.1038/s41598-024-65094-1

**Published:** 2024-06-25

**Authors:** Nasim Bahram Sangani, Jarno Koetsier, Jonathan Mélius, Martina Kutmon, Friederike Ehrhart, Chris T. Evelo, Leopold M. G. Curfs, Chris P. Reutelingsperger, Lars M. T. Eijssen

**Affiliations:** 1 Department of Biochemistry, Cardiovascular Research Institute Maastricht (CARIM), Maastricht University, 6200 MD Maastricht, The Netherlands; 2 GKC, Maastricht University Medical Centre, 6229 ER Maastricht, The Netherlands; 3 DataHub, Maastricht University & Maastricht UMC+, P. Debyelaan 15, 6229 HX Maastricht, The Netherlands; 4 Maastricht Centre for Systems Biology (MaCSBio), Maastricht University, 6200 MD Maastricht, The Netherlands; 5 Department of Bioinformatics - BiGCaT, Research Institute of Nutrition and Translational Research in Metabolism (NUTRIM), Maastricht University, 6200 MD Maastricht, The Netherlands; 6 Department of Psychiatry and Neuropsychology, Research Institute for Mental Health and Neuroscience (MHeNs), Maastricht University, 6200 MD Maastricht, The Netherlands

**Keywords:** Histone deacetylase 6 (HDAC6), Protein–protein interactions, Central nervous system, Alzheimer’s disease, Parkinson’s disease, Amyotrophic lateral sclerosis, Molecular biology, Neuroscience, Literature mining

## Abstract

Due to its involvement in physiological and pathological processes, histone deacetylase 6 (HDAC6) is considered a promising pharmaceutical target for several neurological manifestations. However, the exact regulatory role of HDAC6 in the central nervous system (CNS) is still not fully understood. Hence, using a semi-automated literature screening technique, we systematically collected HDAC6-protein interactions that are experimentally validated and reported in the CNS. The resulting HDAC6 network encompassed 115 HDAC6-protein interactions divided over five subnetworks: (de)acetylation, phosphorylation, protein complexes, regulatory, and aggresome-autophagy subnetworks. In addition, 132 indirect interactions identified through HDAC6 inhibition were collected and categorized. Finally, to display the application of our HDAC6 network, we mapped transcriptomics data of Alzheimer’s disease, Parkinson’s disease, and Amyotrophic Lateral Sclerosis on the network and highlighted that in the case of Alzheimer’s disease, alterations predominantly affect the HDAC6 phosphorylation subnetwork, whereas differential expression within the deacetylation subnetwork is observed across all three neurological disorders. In conclusion, the HDAC6 network created in the present study is a novel and valuable resource for the understanding of the HDAC6 regulatory mechanisms, thereby providing a framework for the integration and interpretation of omics data from neurological disorders and pharmacodynamic assessments.

## Introduction

Histone deacetylase (HDAC) proteins are essential components of the epigenetic machineries that regulate gene expression through post-transcriptional modifications of histones, thereby altering DNA accessibility for transcriptional complexes. The HDAC family encompasses 18 members in humans that catalyze the removal of acetyl groups from lysine residues^1^. Amongst them, histone deacetylase 6 (HDAC6) is a special case as it mainly resides in the cytoplasm and thus also targets numerous non-histone proteins, enabling this protein to function beyond its involvement in transcriptional regulation^2^. HDAC6-mediated pathways are actively involved in a variety of biological processes including cell proliferation and motility^3^, microtubule-mediated transport^4^, memory formation and synaptic plasticity^5^, viral infection^6^, apoptosis^7^, aggresome formation and clearance^8^, and inflammatory responses^9^.

Given this diversity in its functions, HDAC6 has been implicated in various disorders. These extend from different types of cancers such as ovarian, breast, bladder, and thyroid cancer to disorders of the immune system and central nervous system (CNS) including Alzheimer’s disease (AD), Parkinson’s disease (PD), Huntington’s disease, Amyotrophic Lateral Sclerosis (ALS), Charcot-Marie-Tooth disease, and Rett syndrome^10,11^.

HDAC6 exerts its regulatory effect via protein–protein interactions (PPIs), and insights into these regulatory interactions may aid the future development of HDAC6-targeted therapies to treat neurological disorders^12^. PPI databases such as the Biological General Repository for Interaction Datasets (BioGRID) and the intAct molecular interaction database (IntAct) are the primary resources that provide information on PPIs^13,14^. Notably, HDAC6 targets provided by such databases cover the interactions taking place across all different tissues, and given the large heterogeneity in protein expression among the different tissues and cell types, it is not clear whether they all interact or co-express with HDAC6 in the same manner in the CNS. As a complementary approach, the direct evaluation of the literature can support the assessment of tissue-specific binding behavior.

In the present study, we therefore aimed at collecting HDAC6 protein targets that are experimentally validated and reported in CNS or have an impact on neurological disorders. In order to systematically extract and analyze HDAC6 interactions in the CNS, a semi-automated literature screening technique was applied, and meaningful interactions were visualized within a protein interaction network. The constructed literature-based protein interaction network was subsequently compared to a database-derived PPI network and used to visualize and contextualize AD-, PD-, and ALS-associated gene expression alterations.

## Results

### Article retrieval and screening

**Figure 1:**
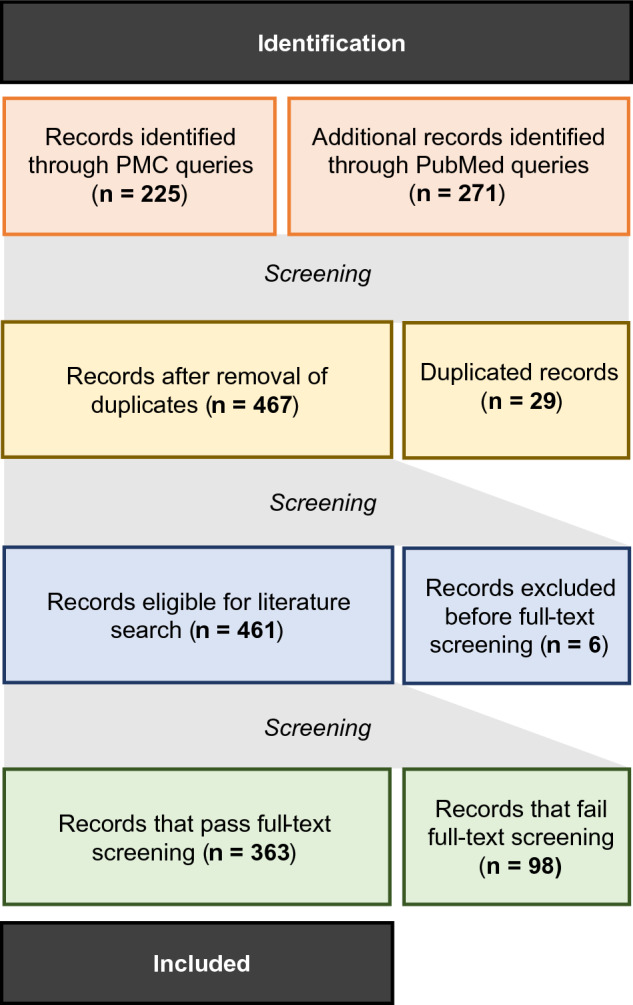
PRISMA Flow Diagram of the search strategy and selection. Please note that the records excluded before full-text screening included articles that were not written in English (n = 2) and for which the full text was not available (n = 4). The records removed after full-text screening included studies not studying HDAC6 function in CNS (n = 98).

In order to create a CNS-specific protein network for HDAC6 interactions based on the current literature, PubMed and PubMed Central (PMC) were queried with defined keywords, resulting in 496 articles of which 467 potential articles remained after screening and removal of the duplicates (Fig. 1). In addition, six more articles were removed because the article was not written in English or the full text was not available. Following further full-text screening, another 98 studies were excluded since they were not studying HDAC6 function in CNS. Eventually, 363 articles remained that met the requirements for this study.

### HDAC6 literature-based network structure and parameters

**Figure 2:**
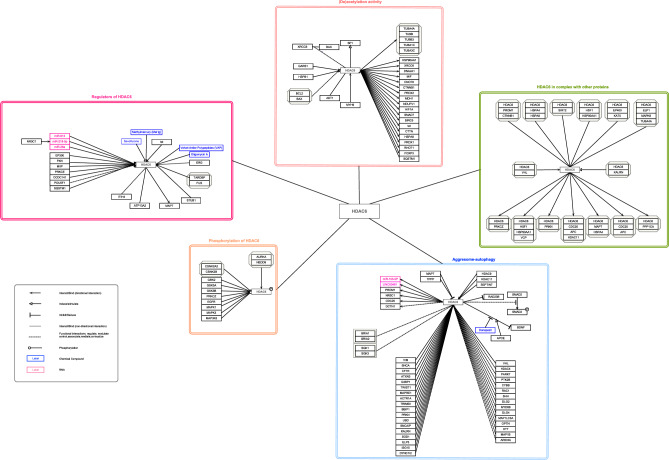
Protein–protein interaction network of HDAC6 identified in CNS. The interactions were derived from the literature using a semi-automated literature screen and were classified into five compartments based on the reported type and function of the interaction. The grey octagonal boxes represent protein complexes. The interactive pathway is available on Zenodo (10.5281/zenodo.10082264) as well as WikiPathways under the accession number WP5426.

A named entity recognition procedure was applied to collect HDAC6 protein interactions from the retrieved articles for the construction of a literature-based HDAC6 protein interaction network. Our literature-based HDAC6 network includes 115 unique genes that share a direct interaction with HDAC6 in the CNS (Fig. 2). In addition to identifying HDAC6 targets, the network encompasses several subnetworks, each assigned based on the nature of the interaction. Specifically, the total of 115 interacting protein partners was categorized into five distinct subnetworks as follows: (1) *(De)acetylation subnetwork*, if HDAC6 deacetylates or is (de)acetylated; (2) *Phosphorylation subnetwork* to represent protein kinases that activate HDAC6 acetylation function; (3) *Protein complex subnetwork* demonstrating protein complexes in which HDAC6 is one of the components; (4) *Regulatory subnetwork* that modulates HDAC6 mRNA or protein expression levels and (5) *Aggresome-autophagy subnetwork* where HDAC6, as an essential component, regulates aggresome formation, transport, and clearance. HDAC6 is displayed as the central hub protein with all interacting partners connected to this central hub with edges presenting the type and direction of the interaction. It is also noteworthy to mention that in some cases the isoenzymes or subunits of the protein found in the study were not specified. Where applicable, such information was retrieved from, *e.g.*, the utilized antibody mentioned in the original research paper. Examples of such proteins were Dynactin (DCTN1), Protein Phosphatase 1 (PPP1CA), and Dynein protein (DYNC1I2). The final HDAC6 pathway is available on Zenodo (10.5281/zenodo.10082264) as well as under the WikiPathways accession number: **WP5426**.

### Identifying potentially overlooked interactions in the HDAC6 network

To identify potentially missing interactions in our literature-based HDAC6 network, we queried IntAct^14^ and BioGRID^13^ databases for HDAC6 protein partners. In total, 53 proteins in our literature-based HDAC6 networks were also reported in at least one of these PPI databases, while 62 proteins were unique to our literature-based HDAC6 network (Fig. S1). Moreover, 212 HDAC6-protein interactions reported in at least one of the queried PPI databases were not present in our literature-based HDAC6 network.

Due to absent or low expression of proteins in the CNS, part of these 212 HDAC6-proteins interactions will not occur in the CNS. Particularly, only 135 of these 212 interactors had a medium or high protein expression in at least one of the reported brain areas according to the Human Protein Atlas database^15^ (proteinatlas.org, version 23.0) (Fig. S2 and Table S1). These 135 proteins encompass potentially missed HDAC6-protein interactions in our HDAC6 network that warrant further investigations.

### HDAC6 protein binding information

**Figure 3:**
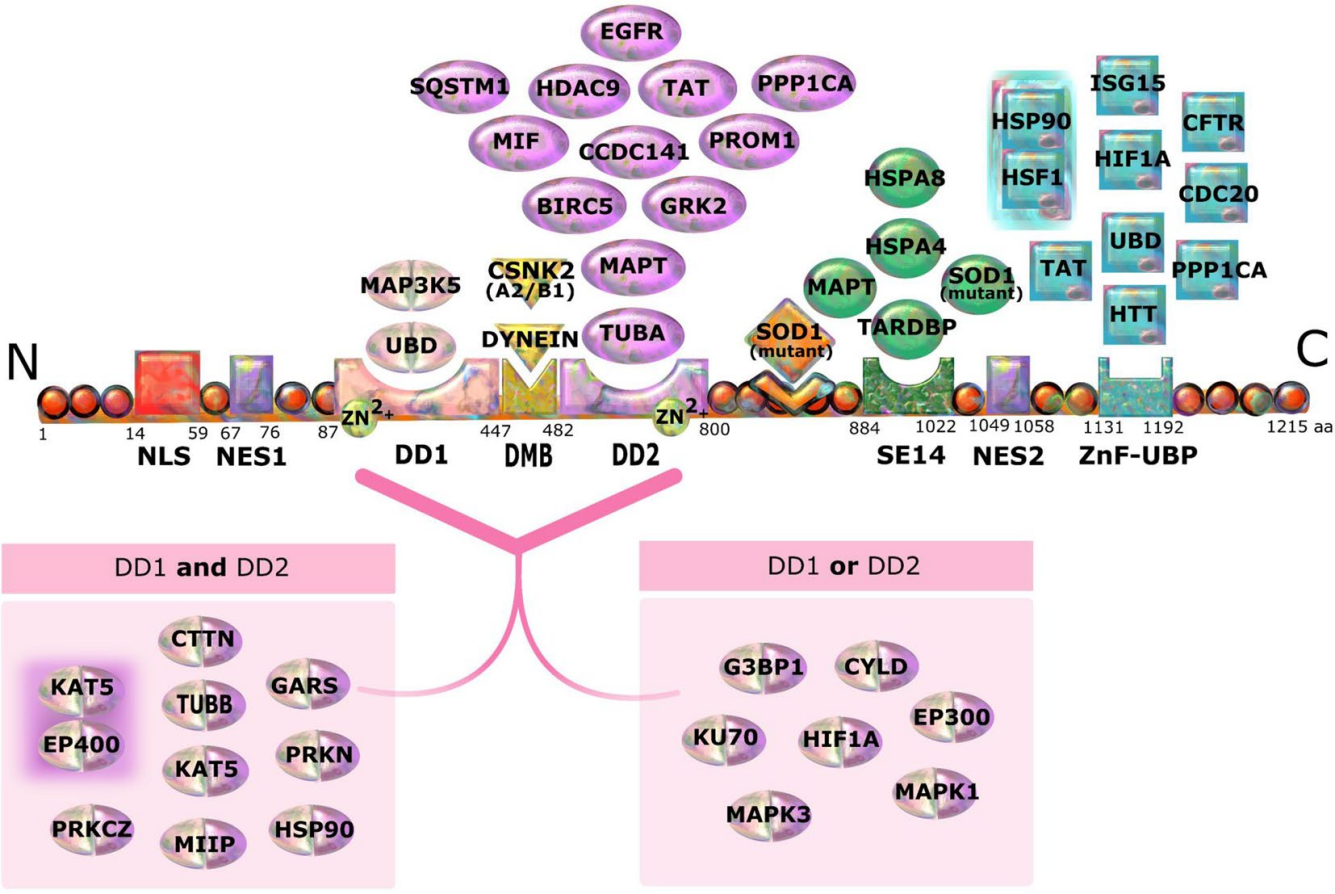
Schematic representation of HDAC6 domains and their protein targets with the indication of their binding sites. From N-terminal; Nuclear localization Signal (NLS), Nuclear Export Signal 1 (NES1), Deacetylase Domain 1 (DD1), Dynein Motor Binding (DMB), Deacetylase Domain (DD2), Ser-Glu-containing tetradecapeptide (SE14) repeat domain, Nuclear Export Signal 2 (NES2), and zinc-finger ubiquitin binding domain (ZnF-UBP), also known as Binder of Ubiquitin Zinc Finger (BUZ). To simplify the figure, subtypes of Alpha and Beta tubulin proteins are not visualized; Alpha and Beta tubulins are visualized as TUBA and TUBB respectively. Similarly, dynein isoforms, DYNLL1 and DYNC1H1, are shown both as Dynein. MAPK3 protein, also known as ERK-1, binds to phosphorylation sites on HDAC6 at threonine 1031 and serine 1035 which are close to SE14 domain. SOD1 was identified to interact with HDAC6 between DD2 and SE14. Besides individual proteins exhibited here, there are also two protein complexes, HSP90-HSF1 and KAT5-EP400 presented with their binding sites in Znf-UBP and DD1 and DD2 domains, respectively. aa, amino acid.

Besides the protein interactions, many studies also investigated the exact binding sites of the protein targets on HDAC6. HDAC6 has multiple functional domains (Fig. 3). These domains include the (catalytic) deacetylase domains 1 and 2 (DD1 and DD2), a zinc-finger ubiquitin binding domain (ZnF-UBP), also known as binder of ubiquitin zinc-finger (BUZ); Ser-Glu-containing tetradecapeptide (SE14) repeat domain; Nuclear Export Signal (NES); Nuclear Localization Signal (NLS); and Dynein Motor Binding domain (DMB). In total, from the 115 proteins that are displayed in the HDAC6 network, the binding site of interaction was identified for 42 proteins. Results indicate that, not surprisingly, the majority of interactions are taking place at the deacetylase catalytic domains either DD1 or DD2, however, some substrates can bind to both domains (Fig. 3). Proteins reported to specifically interact with DD2 include PROM1 (CD133)^16^, CCDC141 (CAMDI)^17^, SQSTM1 (p62)^18^, HDAC9^19^, BIRC5 (survivin)^20^, MIF^21^, and EGFR^22^. While proteins such as HIF1A^23^, HSP90^24^, TAT^25^, UBD (FAT10)^26^, and PPP1CA (PPI)^27^ were described to bind multiple active sites on HDAC6 including both DDs and ZnF-UBP domain. For instance, MAPT interacts with DD2, which mediates MAPT (TAU) deacetylation, and the SE14 domain, for facilitating the aggresome formation^28,29^. The HDAC6 interacting protein binding sites with their references are included in Table S2.

### HDAC6 inhibition targets

**Figure 4:**
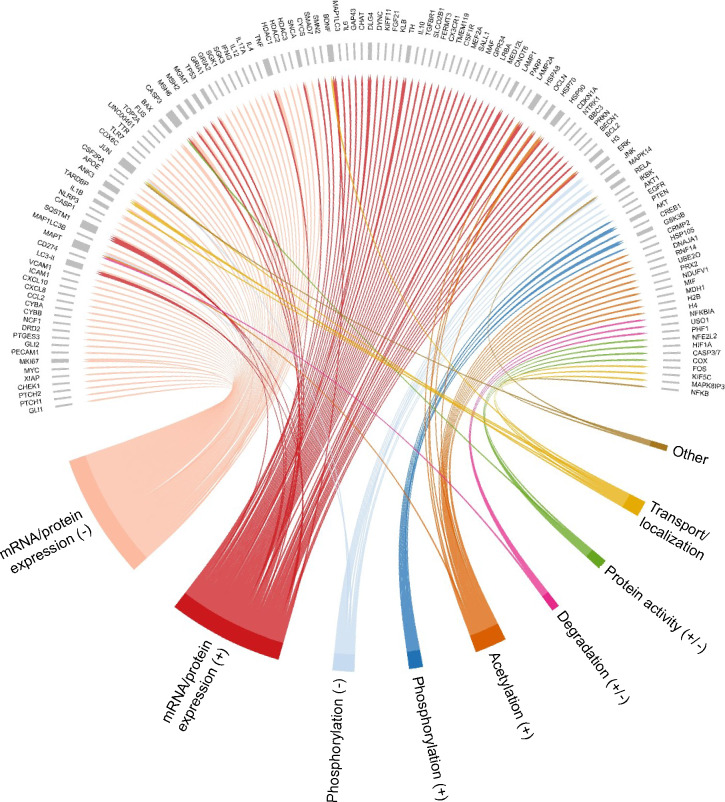
Chord diagram of the reported alterations in response to HDAC6 inhibition. In this diagram, each link represents a reported alteration in the expression levels, post-translational modification, degradation, activity, or transport of the corresponding protein/gene after HDAC6 inhibition. The reported alterations in mRNA/protein expression and phosphorylation levels were further divided into increased ( +) and decreased (−). Note that “other” associations (colored in brown) include changes in DNA binding and promoter activity.

Among the collected articles, a considerable number of articles investigated the therapeutic effects of HDAC6 inhibition in various neurological conditions. It is important to highlight that, while a significant number of targets have been identified, the majority of them are not direct partners of HDAC6. However, HDAC6 knockdown, or its inhibition resulted in alterations in, for example, their expression levels, biological functions, or localization. In total, there were 132 indirect targets identified of which the majority show altered mRNA or protein expression levels in response to HDAC6 inhibition (Fig. 4 and Table S3). Also, various other alterations were reported, such as post-translational modifications and changes in the protein’s activity, localization, or degradation pattern.

### Integration with disease data

As use cases, the literature-derived CNS HDAC6 network was integrated with gene expression profiles of three common brain-related disorders: Alzheimer’s disease (AD) (GSE36980)^30^, Parkinson’s disease (PD) (GSE20292)^31^, and amyotrophic lateral sclerosis (ALS) (GSE76220)^32^. Such approach demonstrates the value of our network as a platform to further identify HDAC6 protein interactions that are potentially involved in the disease pathology. Furthermore, in contrast to using a database-derived PPI network, our HDAC6 network is CNS-specific and clearly categorized to allow for the identification of subnetworks where disease-associated transcriptomic alterations are predominant.

**Figure 5:**
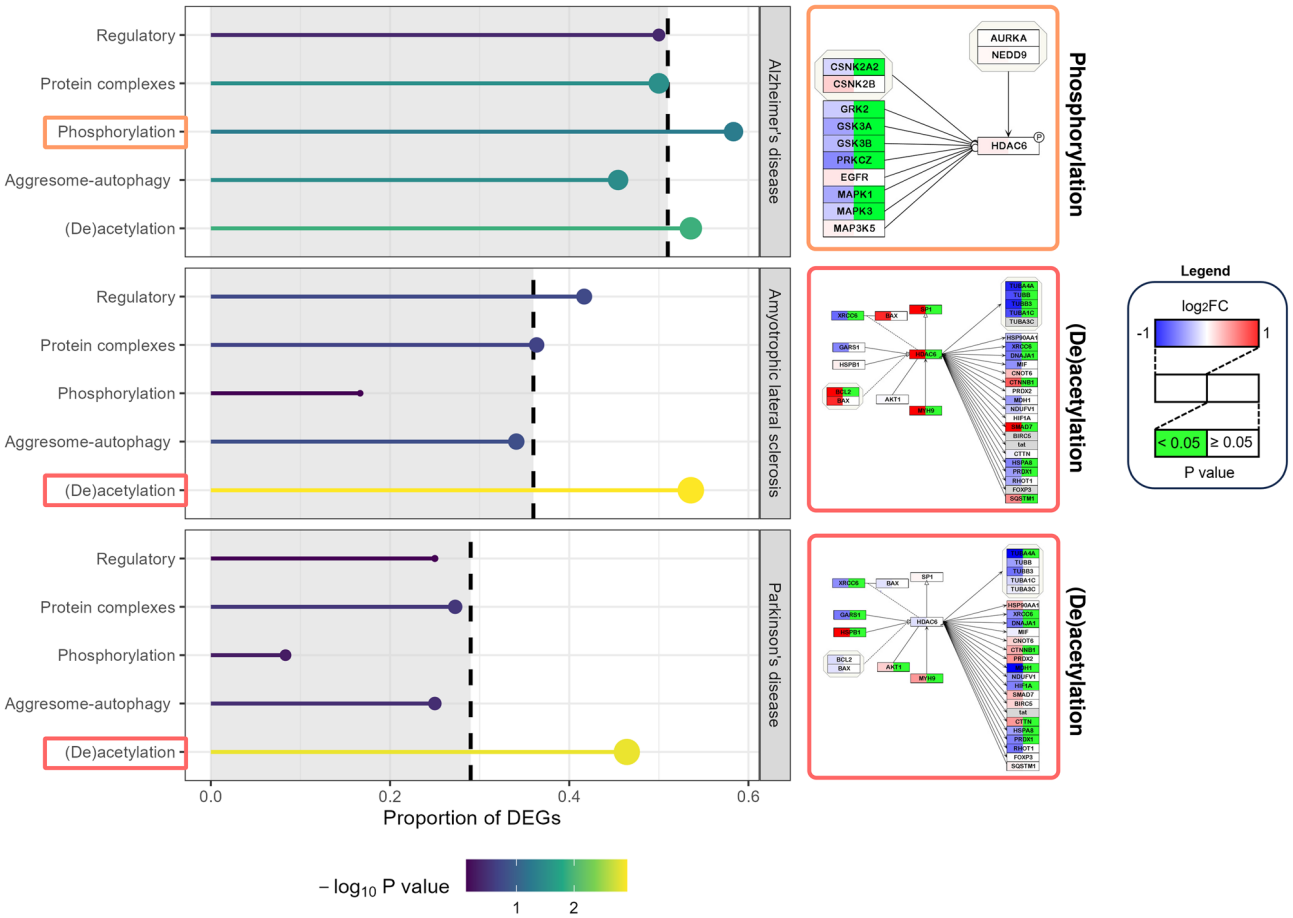
Alzheimer’s disease (AD)-, amyotrophic lateral sclerosis (ALS)-, and Parkinson’s disease (PD)-associated alterations in the HDAC6 subnetworks. The proportion of differentially expressed genes (DEGs) is shown for the different subnetworks in the AD (top panel), ALS (middle panel), and the PD (bottom panel) datasets. The dashed lines indicate the proportion of DEGs in the full HDAC6 network (*i.e.,* all subnetwork combined). The colors and sizes of the data points correspond to the statistical significance of the Fisher’s exact test (*i.e.,* − log_10_ *P* value). For each panel, the mapped expression data is shown for the subnetwork with largest proportion of DEGs (righthand side).

In the AD, ALS, and PD datasets, we identified 51, 36, and 29 differentially expressed genes (DEGs), respectively, in our HDAC6 network. Although HDAC6 itself was not differentially expressed in the AD and PD datasets, it was found to be significantly upregulated in ALS (*P* value = 0.013, log_2_FC = 1.0). The DEGs of the AD, ALS, and PD datasets were all found to be significantly overrepresented in the literature-based network (Fisher’s exact test *P* value = 2.2∙10^–5^, 0.010, and 0.024, respectively). Interestingly, for all three disorders, the changes predominantly occur in the *(de)acetylation subnetwork* (Fig. 5). For instance, significant downregulation of at least one of the microtubule genes (*e.g.,* TUBA4A) was observed in the three datasets (Figs. S3, S4 and S5). However, only in AD, the *phosphorylation subnetwork* was also found to be highly enriched with DEGs (Fig. 5). Particularly, we found that seven genes involved in the phosphorylation of HDAC6 were significantly downregulated in the AD dataset (*i.e.,* CSNK2A2, GRK2, GSK3A, GSK3B, PRKCZ, MAPK1, and MAPK3).

## Discussion

The HDAC6 protein participates in multiple biological processes of which several have implications in neurological disorders. HDAC6-targeted therapy could consequently be beneficial in mitigating the disease phenotype. In the present study, we therefore characterized the role of HDAC6 within the central nervous system. The visualization of the individual protein interactions along with the categorization of these interactions, has offered a more comprehensive perspective on both the upstream regulators and downstream targets of HDAC6.

### The literature-based HDAC6 interaction network

#### Aggresome-autophagy subnetwork

The majority of protein interactions are gathered into the aggresome-autophagy subnetwork, highlighting the fact that the most reported function for HDAC6 is the involvement in the degradation of misfolded and aggregated proteins. To eliminate protein aggregates, cells employ various proteolytic mechanisms such as molecular chaperone system, ubiquitin–proteasome system (UPS), and autophagy which itself is further divided into microautophagy, chaperone-mediated autophagy (CMA), and the aggresome-autophagy, also known as macroautophagy^33,34^. Several lines of evidence, as also represented by the network, point out the crucial role of HDAC6 in the step-by-step process of autophagy, from aggresome recognition to transport and clearance^8,34–36^.

In response to increased levels of misfolded proteins, HDAC6 is translocated to aggresomes^8^ and selectively binds to polyubiquitinated proteins to facilitate their transport for aggresome formation^37^. In fact, HDAC6 acts here as a bridge that links polyubiquitinated proteins (via its BUZ domain) to the dynein motor complex (via its dynein binding domains)^8,34^. Furthermore, HDAC6’s catalytic domains are equally important due to their interaction with microtubule tracks^36^. Through interaction with microtubules, HDAC6 facilitates the autophagic degradation and clearance of several protein aggregates including HTT^36^, LC3^36^, ATXN3^34^, CFTR^8^, KALRN (kalirin-7)^38^, SNCAIP^38^, MAPT^29^, the aggresome marker VIM^39^, and E3- ligase proteins STUB1 (CHIP)^40^, and TRIM50^41^. Moreover, HDAC6 also tightly forms a complex with PRKN (Parkin), a PD-related protein, and under proteasome dysfunction, mediates the bidirectional transport of this complex on microtubules towards aggresomes^42^.

In addition, HDAC6 is required for maturation and proper formation of autophagosomes. This includes the deacetylation of the CTTN (cortactin) protein and its recruitment to aggresomes, a process that is necessary for the fusion of autophagosomes to lysosomes^43^. As a consequence, HDAC6 deficiency was shown to impair autophagosome maturation and the subsequent buildup of autophagosomes and protein aggregates which eventually leads to neurodegenerative phenotypes^43^. HDAC6 also indirectly regulates autophagy through its interaction with the components of the other degradation pathways, such as UPS and CMA. For instance, through its interaction with valosin-containing protein (VCP), HDAC6 has been shown to be an essential regulator of the fate of ubiquitinated proteins, promoting either proteasomal degradation or aggresome formation^35,37^.

#### (De)acetylation subnetwork

In addition to tubulin (*i.e.,* the heterodimeric building block of microtubules) as a well-characterized HDAC6 target, there is a wide subset of other non-histone proteins that are subjected to deacetylation by HDAC6. Depending on the nature of the target protein, deacetylation by HDAC6 can have different effects. As represented by the network, deacetylation by HDAC6 regulates the function of a wide spectrum of protein classes, such as transcription factors, structural proteins, metabolic enzymes as well as protein kinases. HDAC6, therefore, regulates a diverse range of key cellular processes, including gene regulation through targeting transcription factors (*e.g.,* HIF1A^23^, SMAD7^44^, and SP1^45,46^) and protein folding through deacetylation of the chaperone protein family (*e.g.,* HSP90^35,40^, HSPB1^47^, and HSPA8 (HSC70)^28^). HDAC6 is also involved in the immune response through its substrates such as FOXP3^48^, MIF^21^, HSF1^35^, and autophagy regulation by PRKN^42^ and SQSTM1 (p62) ^18^ as well as oxidative stress (PRDX1/2)^49^, DNA repair (XRCC6 (KU70))^50^, and apoptotic processes (BAX, BCL2, and BIRC5)^19,50^.

Besides the role of acetylation in regulating normal cellular activity, there has been a plethora of studies indicating the involvement of HDAC6 in tumorigenesis and the development of cancer cells^10^. Likewise, HDAC6 inhibition was shown to reduce cell invasion and migration in human malignant neuroblastoma^51^ and promote BAX-dependent cell death in neuroblastoma cells through the disassembly of the XRCC6 protein from BAX^50^.

#### Regulatory subnetwork

The regulatory subnetwork encompasses proteins regulating HDAC6 expression and/or activity. HDAC6 is subjected to regulations at the mRNA levels and at the protein level through the modulation of its catalytic domains. For example, TARDBP, in a complex with FUS, positively regulates the HDAC6 mRNA expression levels^52^. Moreover, regulation at the protein level is mediated by the transcription factor EP300, which acetylates the HDAC6 catalytic domains with the subsequent reduction in HDAC6 deacetylase activity^45^. Similarly, several other proteins such as MIIP (IIp45)^53^, CCDC141^17^, and SQSTM1^18^ also bind to the deacetylase domains on the HDAC6 protein to inhibit its activity. Interestingly, the protein ligase STUB1 (CHIP) can bind to the second catalytic domain to ubiquitinate HDAC6 and regulate its half-life. The cellular localization of HDAC6 can also be regulated by other proteins. For instance, APOE ε4 and PRKCE (PKCε) have been shown to positively and negatively regulate the nuclear import of HDAC6, respectively^54^.

#### Phosphorylation subnetwork

The phosphorylation subnetwork includes the protein kinases that mediate HDAC6 activity through phosphorylation. For instance, HDAC6 phosphorylation by the serine/threonine kinases MAPK1 (ERK2) and MAPK3 (ERK1) promotes cell motility via increasing the deacetylase activity, resulting in a reduction in α-tubulin acetylation levels^22^. Conversely, the tyrosine kinase EGFR has the opposite effect on HDAC6 deacetylase activity, leading to an increase in acetylated α-tubulin^55^. HDAC6 is also a substrate for phosphorylation by PRKCZ (PKCζ) which, in addition to its HDAC6 phosphorylation, is also present in an HDAC6 protein complex with SQSTM1, thereby mediating protein aggregates clearance^56^. Likewise, HDAC6 phosphorylation by CSNK2 (CK2) regulates the formation and clearance of aggresomes^56,57^. GSK3B is another protein kinase that phosphorylates HDAC6, a function that mediates mitochondrial transport in hippocampal neurons^58^. Moreover, both GSK3A and GSK3B are suggested to counteract inflammatory tolerance via HDAC6^59^. Finally, besides its impact on HDAC6 deacetylase activity, phosphorylation can also prevent HDAC6 degradation. For instance, MAP3K5 (ASK1), a serine/threonine kinase from the mitogen-activated kinase family, binds to and phosphorylates HDAC6 under hyperoxia condition, an action that prevents HDAC6 ubiquitination by VHL protein resulting in its stabilization and constantly increased expression levels^60^.

#### Protein complex subnetwork

Forming complexes with other proteins enables HDAC6 to extend its function in diverse and distinct pathways which goes beyond its function in the aggresome-autophagy pathway. This includes the regulation of dendritic morphology and stem cell proliferation by forming a complex with ubiquitin ligase CDC20 and APC^61^. Moreover, HDAC11, in a coordinated action with HDAC6 and CDC20 controls dendritic development in immature neurons^62^. Furthermore, in the nucleus, HDAC6 has been found to interact with the chromatin remodeling complex KAT5-EP400 (TIP60-P400) to promote its binding to target genes, a process essential for cellular proliferation^63^. Interestingly, such association was not observed in differentiated cells indicating that upon cell differentiation, HDAC6 re-localizes to cytoplasm and segregates from the complex^63^.

### HDAC6 protein domain interaction

HDAC6 exhibits diversity in its interactions due to the multi-domain structure. In this study, we collected and visualized the interaction sites for 42 binding partners of HDAC6 (Fig. 3). Besides deacetylase and BUZ domains, human HDAC6 (hHDAC6) possesses an SE14 domain, a unique tetradecapeptide repeat sequence, which is absent in mouse, rat, drosophila, and *C. elegans*. Furthermore, hHDAC6 contains intrinsic motifs with nuclear import and export activities. This indicates that compared to the murine ortholog, hHDAC6 deploys different regulatory mechanisms, particularly SE14 and the second nuclear export signal, NES2, for its cytoplasmic retention^64^.

In contrast to other members of the HDAC family, HDAC6 has two catalytic domains, deacetylase domain 1 and 2 (DD1 and DD2). While one catalytic domain is believed to be an internal duplicate of the other one^65^, there are structural differences between them^66^. For instance, although DD1 features a structurally wider active site compared to DD2, it binds to a more limited set of substrates with optimal catalytic activity for those with C-terminal acetyl-lysine residues. In addition, DD1 exhibits significant differences in amino acid substitutions and distinctive conformational variations in conserved residues compared to DD2^66^.

Tubulin, consisting of an α- and a β-subunit, has been one of the first recognized substrates subjected to HDAC6 deacetylation^67^. While numerous studies have investigated the deacetylase activity of HDAC6, there remains a debate regarding the catalytic functionality of the first domain in tubulin and histone deacetylation^65^. Particularly, while some studies suggested that only the second domain is the key catalytic domain^67^, others indicate that both domains act cooperatively^68^ or function independently^65^. Nevertheless, besides tubulin and histone deacetylation, there are several substrates, including MIIP (IIP45)^53^, KAT5 (TIP60)^63^, and G3BP1^69^ for which both HDAC6 domains are required for deacetylation. In addition to its catalytic activity, HDAC6 is involved in the autophagy pathway through its deacetylase-independent function, primarily regulated by its BUZ domain which interacts with both ubiquitinated and non-ubiquitinated proteins^70^.

### HDAC6 interactions in neurological conditions

To reflect on the dynamic status of the network under disease conditions, three gene expression datasets of AD, ALS, and PD were selected from GEO and mapped onto our HDAC6 network. While these publicly datasets have before been analyzed, the mapping on the HDAC6 network gave a novel perspective on these datasets by highlighting the shared and unique transcriptomic alterations among the different HDAC6 subnetworks.

#### Aberrant HDAC6 phosphorylation in Alzheimer’s disease

Although HDAC6 mRNA expression was not found to be differentially expressed in the AD dataset used in the current study, the overexpression of HDAC6 protein levels has previously been described in AD^29^. Interestingly, the upregulation of HDAC6 activity has been reported to be both beneficial, due to its role in tau-aggresome formation^71^, and detrimental, due to its association with tau hyperphosphorylation and the formation of neurofibrillary tangles^29,40^. Moreover, via α-tubulin deacetylation, HDAC6 overexpression reduces microtubule stability, thus disrupting axonal transport^72^.

As shown by the phosphorylation subnetwork, HDAC6 is a substrate for protein kinases such as GRK2, GSK3A, GSK3B, PRKCZ, MAPK1, MAPK3, and CSNK2A2, all of which were found to be downregulated in AD (Fig. 5). In line with these findings, an overall downregulation of protein phosphorylation in AD has previously been reported by Rosenberger et al.^73^. As discussed before, HDAC6 phosphorylation enhances its deacetylase activity, and our results indicate a potential reduction of its deacetylase activity caused by the aberrant expression of protein kinases. If such a hypothesis turns out to be true, then despite the increase in HDAC6 expression level, HDAC6 deacetylase activity is reduced in AD pathology, possibly leading to dysfunctional HDAC6-regulated cellular processes. However, via its BUZ domain, HDAC6 overexpression in AD might still be advantageous due to its role in protein aggregates clearance.

#### (De)acetylation subnetwork in neurodegenerative disorders

The *(de)acetylation subnetwork* was significantly enriched with DEGs of the AD, ALS, and PD datasets (Fig. 5). In this subnetwork, several microtubule genes, which are subject to deacetylation by HDAC6, were downregulated in all three disorders. Indeed, microtubule dysfunction is a common feature among many neurodegenerative disorders, including AD, ALS, and PD^74^. Furthermore, other deacetylation targets of HDAC6, such as HSPA8, DNAJA1, and XRCC6 were also found to be downregulated in all three datasets. Since (de)acetylation is essential for regulating the stability^75^ and function^76^ of proteins, modulation of HDAC6 expression may be beneficial to counteract the observed expression changes in this subnetwork. For instance, tubulin acetylation is associated with increased microtubule stability^77^ and, hence, HDAC6 inhibition might be used to oppose the downregulated expression levels of these microtubule-associated genes.

### HDAC6 inhibitors and their therapeutic potential

The field of HDAC6 inhibitors and their therapeutic effects has seen extensive research and development. However, in the context of neurological disorders, HDAC6 is reported to have seemingly contradictory effects^78^. On one hand, HDAC6 is believed to have detrimental effects as the overexpression of HDAC6 has been shown to cause tubulin and cortactin hypo-acetylation with the subsequent impact on cell motility^79^. This is supported by the observed improvement of axonal transport by HDAC6 inhibitors^4^. On the other hand, HDAC6 is also believed to have neuroprotective effects through the clearance of protein aggregates, primarily via its BUZ domain. For instance, studies have demonstrated that an increase in HDAC6 can suppress tau accumulation, while its deficiency has been shown to accelerate tau pathology and cognitive decline and contribute to learning deficits^5,28^.

In light of these contradictory findings, further investigation is required to fully understand the role of HDAC6 in neurological disorders. This is further emphasized by the fact that despite promising pre-clinical outcomes, no HDAC6 inhibitors have been advanced into clinical trials for neurological disorders^80^. It is also important to consider factors such as the brain region specificity of HDAC6 expression^81^ and the delicate balance between its various cellular functions^78^ when assessing its potential as a therapeutic target in neurodegeneration. For instance, HDAC6 inhibitors have a wide range of molecular effects (Fig. 4) and the integration of our network with the disease transcriptomics data indicated that not all HDAC6 subnetworks are equally altered (Fig. 5). To optimize response and avoid adverse effects, treatments should specifically target those subnetworks affected by the disease. Mapping the transcriptomics and proteomics data from drug screening approaches on our HDAC6 network may guide the identification of promising drug candidates.

### The semi-automated literature evaluation workflow

In this paper, we applied a semi-automated workflow to systematically extract all interaction partners of HDAC6 from the literature and to display these in a digital interactive graphical representation, uploaded to WikiPathways^82^ (wikipathways.org). This process can be beneficial to researchers wishing to collect and represent the interaction partners of other proteins. The strengths of our approach reside in the evaluation of papers by using a stepwise computerized approach, extracting precalculated text-mining results of Europe PMC via its programming interface, followed by a digital evaluation of co-occurrence of gene/protein names and their indicated relationships using a customized script. Europe PMC is an open resource that generates named entity recognition results on all papers in its database, based on the full text for open-access papers and the publicly accessible parts (including title and abstract) for closed papers^83^. These results include, among other annotations, the naming of genes and proteins, as well as gene-disease relationships in all papers. The strength of using their annotations as a first step is twofold: it supports not missing named entities by manual reading of the papers and supports a scale-up of the process to more papers than could be feasibly assessed otherwise. A limitation may be that it cannot search the non-open content of part of the papers. Our two-step approach that extracts all named proteins/genes from the open parts of papers using Europe PMC, and then searches all full papers by a custom script, mitigates this limitation, by still allowing a full-text search for interactions between any of these identified proteins/genes of all papers that the researcher’s institute can access. This means that any information available on the shortlisted genes/proteins will still be found, even in non-open-access content. Only in the case where a protein is named in the closed content of a paper to interact with the protein of interest and that first protein is not named at all in any open part of any other paper, this would be missed. This shall be a very limited part of the information present in the literature. Moreover, with current open-access policies, it is expected to become an even lesser part in the future, again stressing the value of open-access publishing directives.

After extraction of all named interactions in the two-step computerized approach, we manually curate these and represent them in a digital resource, in an interactive visual network-style format uploaded to the WikiPathways database. This allows other researchers to access and investigate this information and use it in their processing workflows for high-throughput (omics-based) or other datasets, to interactively map and visualize these datasets, and perform analyses such as overrepresentation analysis. This enhances the further study and retrieval of new information about the function of proteins, such as in our case HDAC6, and their involvement in physiological and pathological conditions of interest. Nevertheless, it should be noted that the occurrence of post-transcriptional regulatory processes and protein compartmentalization warrant caution when interpreting the findings of transcriptomics and proteomics mapping onto the HDAC6 network.

By making our scripts available, we support other researchers to apply this same semi-automated workflow to systematically collect information on their proteins of interest and help advance science by representing their findings in publicly accessible digital representations, supporting other teams in turn to gain new knowledge on these proteins by mapping and analyzing their generated datasets.

## Conclusion

In summary, our study shows that HDAC6 is one of the main hub proteins in the molecular interaction networks of several neurological diseases. We observed a diversity of proteins with different functions interacting with HDAC6 in the CNS, which enables the protein to be an important modifier in several biological events. The literature-based network created in the present study is more than just a review of the current knowledge as it can act as a novel and valuable resource for omics data analyses of brain-related disorders and the interpretation of their results. Finally, the molecular interaction network provided in this study can as well be useful for assessing the pharmacodynamics of HDAC6-targeted therapy since it provides information about direct and indirect changes in the HDAC6 regulatory network upon treatment. In addition to the valuable insights generated about HDAC6, the semi-automated workflow that we used and shared can be applied to any other protein of interest, facilitating other researchers to systematically obtain results from literature and to represent them in a digitized format, which, in turn, will support others and will advance scientific knowledge.

## Methods

### Systematic review of HDAC6 interactions in CNS

#### Literature retrieval and screening

In order to create a context-specific protein network for HDAC6 interactions, candidate articles were retrieved and downloaded using PMC Open Access (date: 2023–04-12). The query was formulated using a set of Medical Subject Headings (MeSH) terms and general key terms as follows: “Histone Deacetylase 6” OR “HDAC6” AND “Brain”, OR “brain cells” OR “Neurological disorders”, OR “Neurons”, OR “Astrocyte”, OR "Microglia", OR "Glia", OR "Oligodendrocytes" OR “Cortex” OR “Cerebellum” OR “brainstem”. The two terms “HDAC6” and “Histone Deacetylase 6” were used as query words both in the title and in the abstract. In addition, we used similar terms for HDAC6 protein, including HDAC-6, Hdac6, and hdac6.

Furthermore, querying PubMed (title and abstract) and Web of Science with the same keywords also returned a considerable number of (non-open access) articles containing valuable information on HDAC6 protein interactions. These articles were also included in the study. Articles were screened according to the PRISMA guidelines^84^. Inclusion criteria encompassed articles that assessed HDAC6 protein function and/or interactions in the CNS or in relation to neurological conditions and were written in the English language. No restriction was applied concerning the year of publication.

#### Automatic processing of articles

For the named entity recognition, all Europe PMC annotations of the previously selected papers were extracted via the R Client for its RESTful Web Service^83^ (*europepmc* R library version 0.4.3.) by passing their PMC and Pubmed identifiers to the “epmc_annotations_by_id” function. Thereafter, results were filtered to only keep those of type "Gene_Proteins'' or those representing UniProt identifiers participating in a "Gene Disease Relationship”. From these annotations, no distinction was made between genes and their protein products, and they were considered the same entity.

The list of protein/gene names, which was retrieved from Europe PMC after the initial screening, was used to filter the corpus based on sentences containing protein names and their interaction information. The papers were downloaded as pdf files and transformed into text format using *xpdf-tools* ^85^ (version 4.04). Text processing was performed in R 4.3.1^86^ using the packages *stringr*^87^ (version 1.3.1) and *tokenizers*^88^ (version 0.2.1).

#### Manual selection of curated interactions

Information retrieved from sentences contained two parts: the name of and the association between the proteins/genes reported to interact with each other. Although the majority of interactions were found to take place with HDAC6 as one protein of the interacting pair, there were also a significant number of indirect interactions identified to occur when the HDAC6 gene was knocked down using, for example, small interfering RNA (siRNA) or when its activity was inhibited using chemical compounds as inhibitors. The direct HDAC6 protein interactions, with HDAC6 as part of the interaction pair, were used for the creation of the literature-derived interaction network. The indirect interactions that were identified through HDAC6 inhibition were separately collected and categorized.

#### Creation of the literature-based HDAC6 interaction network

PathVisio^89^ (version 3.3.0), an open-source software for pathway visualization and analysis, was used to construct and visualize the direct HDAC6 protein interactions in a machine-readable pathway model. In case literature-extracted interactions, collected from the previous step, contained gene/protein name synonyms, they were replaced by their HGNC-approved gene symbols. HDAC6 protein partners were visualized as data nodes and their interactions with HDAC6 were displayed by directional edges, if applicable, with different styles based on their type of interaction. Further information about each interaction such as the reference of the related article and the sentence from which the information had been extracted was added to “Data Node Properties” of each node in the pathway. The final HDAC6 pathway was uploaded to the public pathway database WikiPathways^82^ (wikipathways.org).

#### Categorization of HDAC6 inhibition interactions

The indirect HDAC6 interactions, as identified via HDAC6 inhibition or knockdown, were manually categorized into one or more of the following modes of action: (1) alterations in mRNA/protein expression levels, (2) protein phosphorylation, (3) protein acetylation, (4) protein activity, (5) protein degradation, (6) cellular transport/localization, and (7) other alterations. Where possible, the direction of effect (*e.g.,* up- or downregulation) was collected as well.

#### Binding domains and target binding sites on HDAC6

Besides the HDAC6-protein interaction, a substantial number of articles also investigated the binding sites of the protein targets on HDAC6. Therefore, in addition to the PPIs, where applicable, we also collected these binding site interactions and visualized them using Inkscape^90^ (version 1.3).

### Identifying potentially overlooked interactions in the HDAC6 network

#### Collection of database-derived PPIs

A PPI network of HDAC6 was constructed from the two widely used and curated PPI databases, BioGRID^13^ and IntAct^14^, to provide an overview of the current PPI knowledge. To retrieve protein interactors from the BioGrid and IntAct databases, the Cytoscape^91^ (version 3.10.1) apps *BioGrid Data Source* (version 3.3.1) and *IntAct App* (version 1.0.0) were used, respectively. The UniProt IDs from the IntAct network were converted to Entrez Gene IDs (in case of non-unique mappings, the Entrez Gene ID with the lowest number was chosen, representing the earlier known gene), after which the union of the BioGRID and IntAct networks’ Entrez Gene IDs was taken to merge the two networks.

#### Mapping of brain protein expression data

Many known HDAC6 protein interactors from PPI databases, which are not yet reported in the context of CNS-related cells or brain tissue, might also have a role in neurological dysfunctions through their interaction with HDAC6. To uncover this potential, we mapped the brain protein expression named “Normal tissue data”, collected from the Human Protein Atlas database^15^ (proteinatlas.org, version 23.0), onto the HDAC6 PPIs from the databases. The dataset comprises protein expression data from human tissues obtained through immunohistochemistry using tissue microarrays based on Ensembl version 109. This dataset includes information such as protein expression levels categorized as high, low, or not detected, along with their localization in various tissues and cell types. The categorization is based on the staining intensity and fraction of stained cells. We excluded the expression categories classified as uncertain which happens in case of exclusive availability of multi-targeting antibodies, low consistency with RNA sequencing data, or dissimilar patterns of paired antibodies. Furthermore, we selected those tissues related to different brain regions: caudate, cerebellum, cerebral cortex, dorsal raphe, hippocampus, hypothalamus, and substantia nigra.

### Pathway validation using neurological disease expression data

#### Data collection

To demonstrate the usefulness of integrating the HDAC6 network with CNS-derived transcriptomics data, we re-analyzed publicly available RNA-sequencing and microarray data from post-mortem CNS tissue of three diseases: AD, PD, and ALS as case studies. The selection criteria for these studies include the availability of raw data (*i.e.,* Affymetrix CEL files or raw count table) in the Gene Expression Omnibus (GEO) database and sampling from disease-relevant post-mortem CNS tissue (*i.e.,* PD: substantia nigra, AD: hippocampus, and ALS: spinal motor neurons).

The dataset GSE36980, created by Hokama et al.^30^, was selected for the gene expression analysis of AD. In the original study, Hokama et al*.* investigated the effect of AD pathology on changes in expression levels using the Affymetrix GeneChip Human Gene 1.0 ST microarray platform. The raw microarray data from the post-mortem hippocampus of advanced AD patients (Braak stage V-VI) and healthy controls were selected for the analysis. Moreover, to study PD, the dataset GSE20292 by Zhang et al*.*^31^ was used. In their study, Zhang et al*.* investigated the alterations in the transcriptomics profile of the post-mortem substantia nigra from neuropathologically confirmed PD patients and pathologically normal controls using Affymetrix Human Genome U133A Array. The raw CEL files were selected for the analysis. Finally, the raw count table of the RNA-seq dataset GSE76220 by Krach et al*.*^32^ was utilized. This dataset included the transcriptomics profiles of post-mortem spinal motor neurons of sporadic ALS patients and non-ALS control patients.

#### Data processing

For the microarray datasets GSE36980 (AD) and GSE20292 (PD), the pre-processing procedure that is available from *ArrayAnalysis*^92^ (https://github.com/arrayanalysis) was performed on the raw data. Subsequently, to identify DEGs, ArrayAnalysis uses the *limma* R/Bioconductor software package^93^ (version 3.50.3) to conduct statistical analysis based on an adapted *t-*test. Probes were annotated with Ensembl gene identifiers using the *biomaRt* R/Bioconductor software package^94^ (version 2.56.1). In the case of multiple probes annotated to the same gene, the one with the lowest *P* value in the differential gene expression analysis was selected as the representative probe.

Additionally, the *EdgeR* R/Bioconductor workflow^95^ (version 4.0.12) was applied for the pre-processing and differential expression analysis of the RNA-seq dataset GSE76220 (ALS). *EdgeR* is specifically designed for RNA-seq experiments and uses the quasi-likelihood F-test to infer statistical significance. Finally, the Entrez Gene identifiers were converted to Ensembl gene identifiers using the *biomaRt* R/Bioconductor software package^94^ (version 2.56.1).

For each of the three datasets, a list of significantly up- and down-regulated genes was obtained based on a cutoff of nominal *P* value < 0.05. The statistical outputs were imported into *PathVisio*^89^ (version 3.3.0) and visualized on our HDAC6 network. Finally, the Fisher’s exact test was applied to assess whether the DEGs were significantly overrepresented in the HDAC6 subnetworks.

### Supplementary Information

Supplementary Figures.

Supplementary Tables.

## Data Availability

The R scripts used in the current study are available on GitHub (https://github.com/jarnokoetsier/HDAC6Network). The stable-version of our CNS-specific HDAC6 interaction network is available on Zenodo (10.5281/zenodo.10082264). The community-curated version of our CNS-specific HDAC6 interaction network is available on WikiPathways under the accession number: WP5426 (https://www.wikipathways.org/pathways/WP5426.html).
