# Supplementary material for: A novel insight into neurological disorders through HDAC6 protein–protein interactions
Source: Sci Rep. 2024 Jun 25;14:14666. doi: 10.1038/s41598-024-65094-1 (PMC11199618; doi:10.1038/s41598-024-65094-1)
Supplement: Supplementary file 1 — Supplementary Figures. [file 41598_2024_65094_MOESM1_ESM.pdf]

Supplementary Information for

A novel insight into neurological disorders through HDAC6 protein-protein interactions

Nasim Bahram Sangani*, Jarno Koetsier, Jonathan Mélius, Martina Kutmon, Friederike Ehrhart, Chris T. Evelo, Leopold M.G. Curfs, Chris P. Reutelingsperger, Lars M.T. Eijssen

*Corresponding author: Nasim Bahram Sangani
Email: nasim.sangani@maastrichtuniversity.nl

This PDF file includes:

- Figure S1 – Page 2
- Figure S2 – Page 3
- Figure S3 – Page 4
- Figure S4 – Page 5
- Figure S5 – Page 6

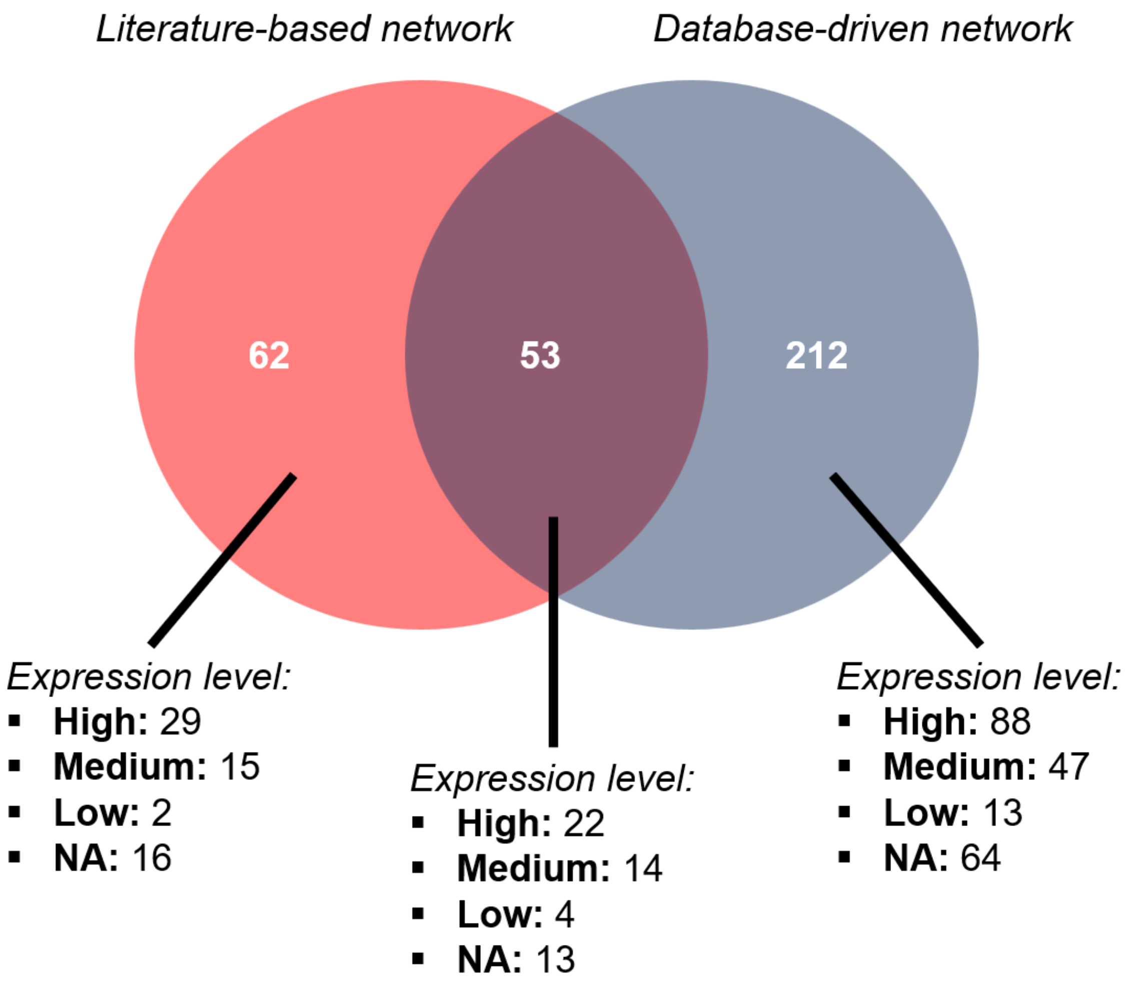

Figure S1 Venn diagram of database-driven and literature-based networks. The brain expression status of the proteins was retrieved from the Human ProteinAtlas (proteinatlas.org)

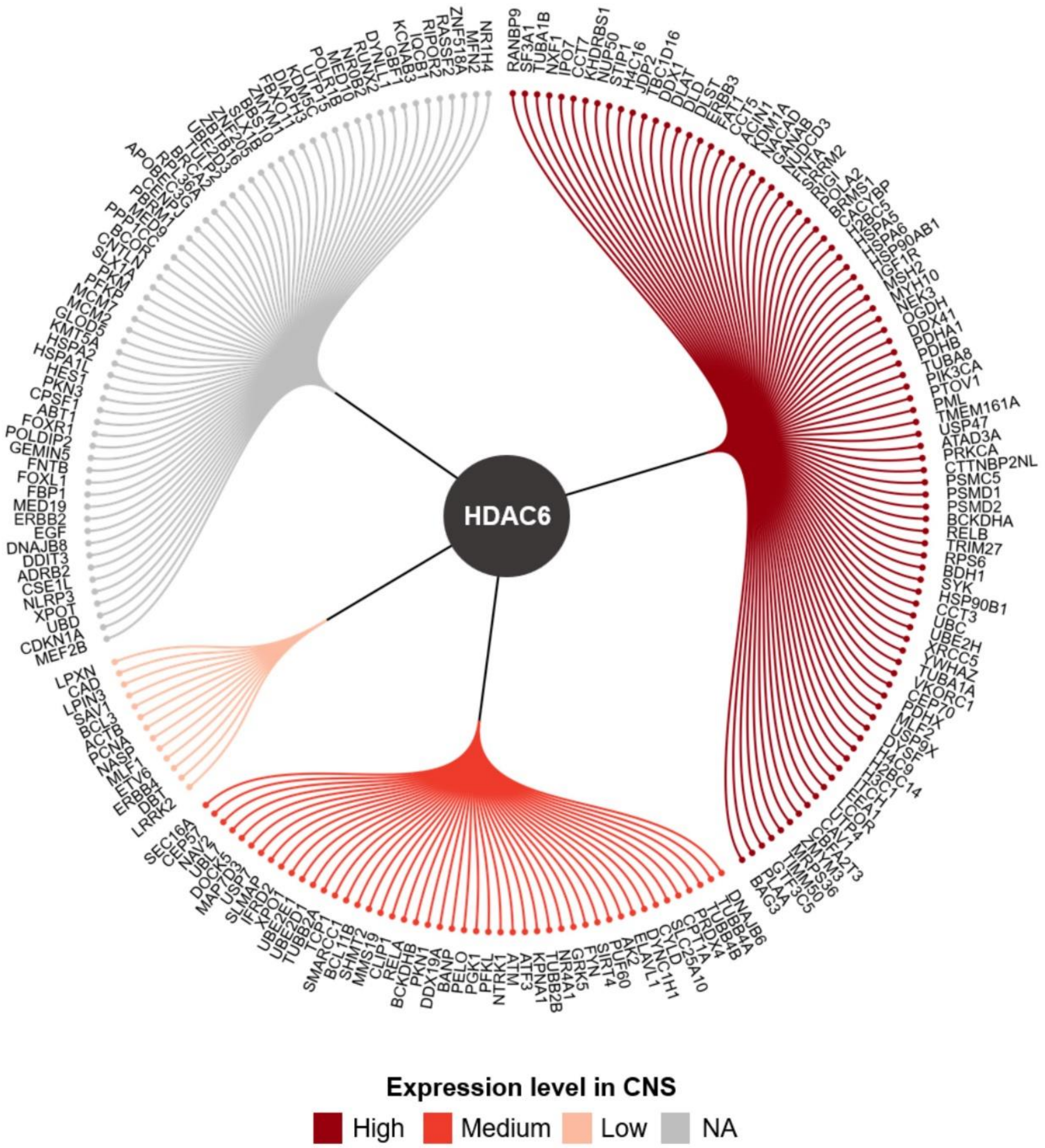

Figure S2 Network diagram of the HDAC6-protein interactions that are potentially overlooked in the literature. The network diagram only includes the HDAC6 interactors that are in the IntAct and/or BioGRID PPI databases but not in the literature-based HDAC6 network. Based on the brain protein expression data from the ProteinAtlas, these proteins are grouped into low (n = 13), medium (n = 47), or high (n = 88) expression classes. 64 proteins had a non-assessable (NA) expression.

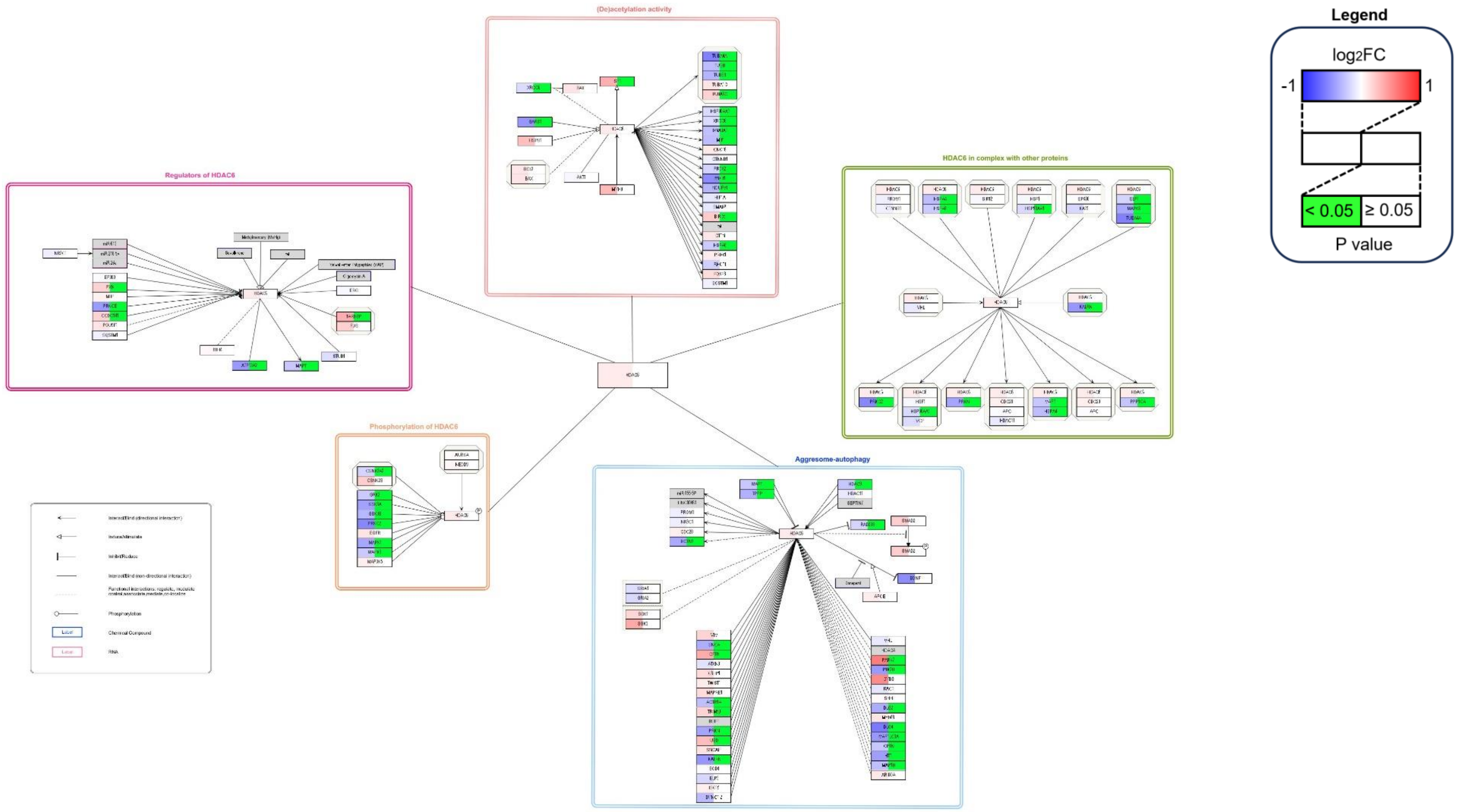

Figure S3 Visualization of Alzheimer's disease (AD) expression data on the literature-derived network. The AD dataset with the accession number *GSE36980* was retrieved from the Gene Expression Omnibus (GEO).

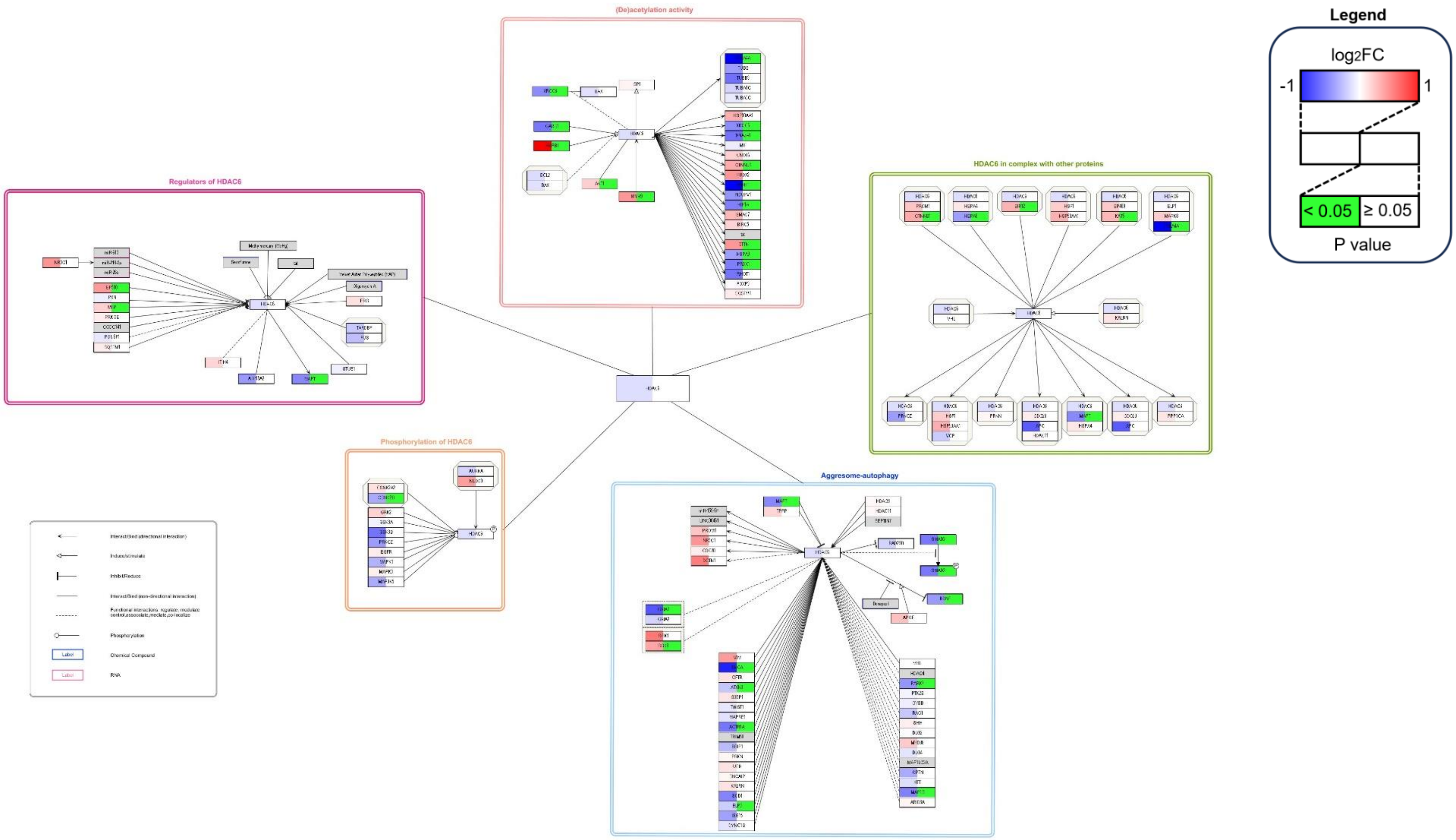

Figure S4 Visualization of Parkinson’s disease (PD) expression data on the literature-derived network. The PD dataset with the accession number *GSE20292* was retrieved from Gene Expression Omnibus (GEO).

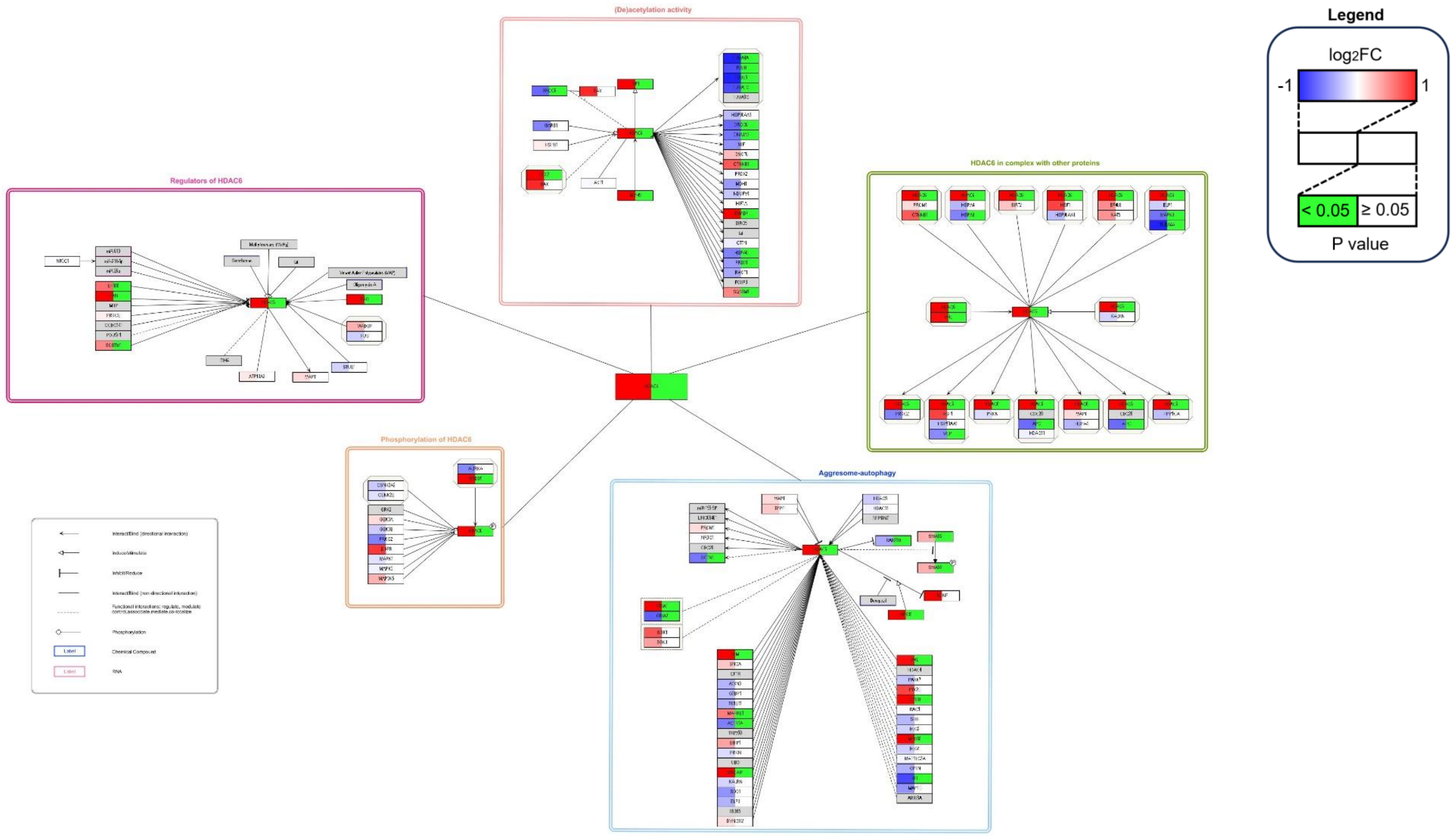

Figure S5 Visualization of amyotrophic lateral sclerosis (ALS) expression data on the literature-derived network. The ALS dataset with the accession number *GSE76220* was retrieved from Gene Expression Omnibus (GEO).
